# Facile synthesis of sewage sludge-derived *in-situ* multi-doped nanoporous carbon material for electrocatalytic oxygen reduction

**DOI:** 10.1038/srep27570

**Published:** 2016-06-07

**Authors:** Shi-Jie Yuan, Xiao-Hu Dai

**Affiliations:** 1 State Key Laboratory of Pollution Control and Resource Reuse, College of Environmental Science and Engineering, Tongji University, Shanghai, 200092, China

## Abstract

Developing efficient, low-cost, and stable carbon-based catalysts for oxygen reduction reaction (ORR) to replace the expensive platinum-based electrocatalysts remains a major challenge that hamper the practical application of fuel cells. Here, we report that N, Fe, and S co-doped nanoporous carbon material, derived via a facile one-step pyrolysis of sewage sludge, the major byproduct of wastewater treatment, can serve as an effective electrocatalyst for ORR. Except for the comparable catalytic activity with commercial 20% Pt/C via a nearly four-electron transfer pathway in both alkaline and acid medium, the as-synthesized co-doped electrocatalyst also exhibits excellent methanol crossover resistance and outstanding long-term operation stability. The organic compounds in sewage sludge act as the carbon source and the *in-situ* N and S dopant in the fabrication, while the inorganic compounds serve as the in-built template and the *in-situ* Fe dopant. Our protocol demonstrates a new approach in the economic and eco-friendly benign reuse of sewage sludge, and also provides a straightforward route for synthesizing excellent carbon-based electrocatalysts as promising candidates for ORR directly from a type of waste/pollution.

The high efficiency and environmental benignity of fuel cells has led to them being considered as one of the most promising energy conversion technologies today^1^,^2^. The cathode oxygen reduction reaction (ORR) is crucial in controlling the performance of fuel cells^3^,^4^. However, the sluggish kinetics, high cost, and poor stability of commercially available Pt-based cathode ORR electrocatalysts, such as 20% Pt/C, has been a major limiting factor in the practical application of the fuel cells^5^,^6^. Much effort has therefore been devoted to exploring efficient, durable, and low-cost alternative ORR electrocatalysts based on precious metal-free and even metal-free materials^7^,^8^.

Recent research efforts have focused on carbon-based materials doped with metal or nonmetal heteroatoms^7^,^8^. Most carbon-based nanomaterials have desirable mechanical and electrical properties, such as large surface areas and high electrical conductivity, making them promising supports and substrates for heteroatom doping. Carbon-based nanomaterials have been used as supports for nonprecious metals, mainly geologically abundant transition metals such as iron and cobalt, to enable high ORR catalytic performance^9^,^10^. Furthermore, metal or nonmetal heteroatom doped carbon nanomaterials, particularly M-doped C (M: Fe or Co) and N-doped C, have also been demonstrated as promising electrocatalysts for ORR due to their pronounced electrocatalytic performance, remarkable methanol tolerance, long-term stability, and relatively low cost. Indeed, many previous studies confirm that binary/multi-doped carbon-based nanomaterials (S-N-C, P-N-C, B-N-C, M-N-C, etc.) can significantly improve the ORR performance of N-doped materials^11^,^12^,^13^,^14^,^15^. Significant progress has been achieved in the synthesis of heteroatoms of doped carbon-based electrocatalysts. Most of the previous doped carbon nanomaterials were derived from multiple hazardous chemicals precursors^7^,^8^, so it is challenging but extremely desirable to develop high activity multi-doped carbon-based nanomaterials from waste or pollutants via facile methods without any activation that are suitable for large-scale production^16^,^17^.

Sewage sludge, the major byproduct of wastewater treatment, is defined as a pollutant by the US Environmental Protection Agency^18^. The increasing amount of sewage sludge generated worldwide, and the imposition of more stringent regulations, means that its disposal and reuse has become a thorny issue in water and sanitation engineering^19^,^20^. The need to develop a method of reusing sewage sludge that is economically, socially, and environmentally sustainable is of particular concern. Sewage sludge mainly consists of organic material, such as bacterial cells, extracellular polymeric substances, and cellulose, and inorganic compounds in the form of various oxides and salts, and a wide variety of heavy metals. Sludge pyrolysis can convert approximately half of the organic matter in sewage sludge into renewable liquid fuels and chemical feedstock, and immobilize the remainder and the inorganic matter into a stabilized form of pyrolytic residue (biochar)^21^,^22^. This residue, a carbonaceous matrix byproduct containing various heteroatoms (N, S, Fe, etc.), is generally recognized as an alternative adsorbent^23^.

In this study we demonstrate the synthesis of multi-doped nanoporous carbon materials, as efficient electrocatalysts for ORR in both alkaline and acid medium, from sewage sludge via a facile, low-cost, and scalable pyrolysis approach without any activation. The organic matter in sewage sludge, some of which is abundant in nitrogen and sulfur, is exhibited as the structure-directing substance and the *in-situ* N, S dopant precursor during this pyrolysis process. The iron compounds in sewage sludge can function as both the catalyst, increasing the degree of graphitization, and the source of iron for *in-situ* Fe-doped. Other special inorganic compounds in the sewage sludge, particularly the SiO_2_, act as an in-built template, which prevents agglomeration and results in the formation of unique porous structures. The sewage sludge-derived *in-situ* multi-doped nanoporous carbon material, with a high specific surface area and numerous heteroatoms, presents remarkably high ORR activity, excellent ability for immune methanol crossover, and superior durability in both alkaline and acid medium. Our protocol reveals a new approach for the value-added reuse of sewage sludge, and confirms its true potential as a candidate for the synthesis of multi-doped nanoporous carbon material as a highly active ORR catalyst.

## Results

### Textural properties of the as-synthesized catalysts

**Figure 1:**
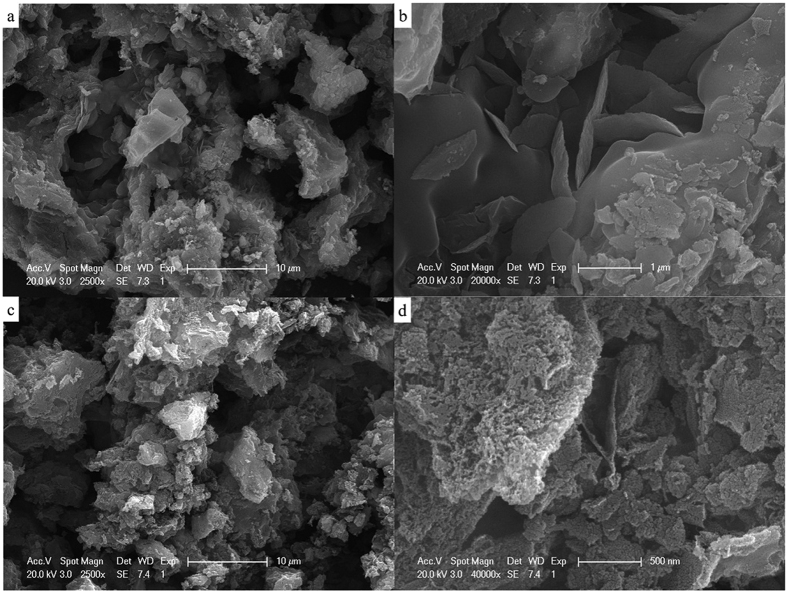
SEM images of the as-prepared SS-800 (**a,b**) and SS-AW (**c,d**) at different magnification.

The surface morphology and composition of the as-synthesized nanoporous carbon material were examined using SEM-EDX. The typical images are shown in Figs 1, S2 and S3. Porous structures and complicated composition were observed both for SS-800 and SS-AW. The specific porous structure of SS-800 (Figs 1a,b, and S2) was formed during the pyrolysis process, with the pyrolysis and the carbonization of the organic matter in the sewage sludge occurring simultaneously. The inorganic content in the sewage sludge, particularly the special component SiO_2_, acted as the in-built template during the synthesized process preventing agglomeration. This resulted in the formation of mesoporous structures with complicated compositions (Figs 1a,b, and S2). The obvious Fe and S content observed in the SS-800 suggests that the *in-situ* self-doped heteroatom of this sewage sludge-derived nanomaterial, with the special component of Fe and S compounds innate in the sewage sludge as an ideal precursor and nature dopant (Figure S2 and Table 1). Many sheets, which were mixture of the C, Ca, Mg, Al, Si, and so on (Figure S2a), can be observed (Fig. 1b). Compared with SS-800, SS-AW displayed rougher hierarchical-pore structures with the disappearance of these sheets (Fig. 1c,d). These sheets were destroyed along with the increase in porosity during the acid washing step, which partially removes the inorganic compound and decreasing the ash content (Figure S2b). This also increased the surface area of the catalyst. This increase was further evident by the increased BET surface area of the SS-AW, compared with that of the SS-800 (Table 1). The continued presence of Fe and S content in the SS-AW (Figure S3 and Table 1) confirmed the possible Fe and S self-doped heteroatom of this sewage sludge-derived nanomaterial. TEM images of the as-synthesized catalyst further reveals specific nanoporous structure of the as-synthesized catalyst (Figure S3).

**Figure 2:**
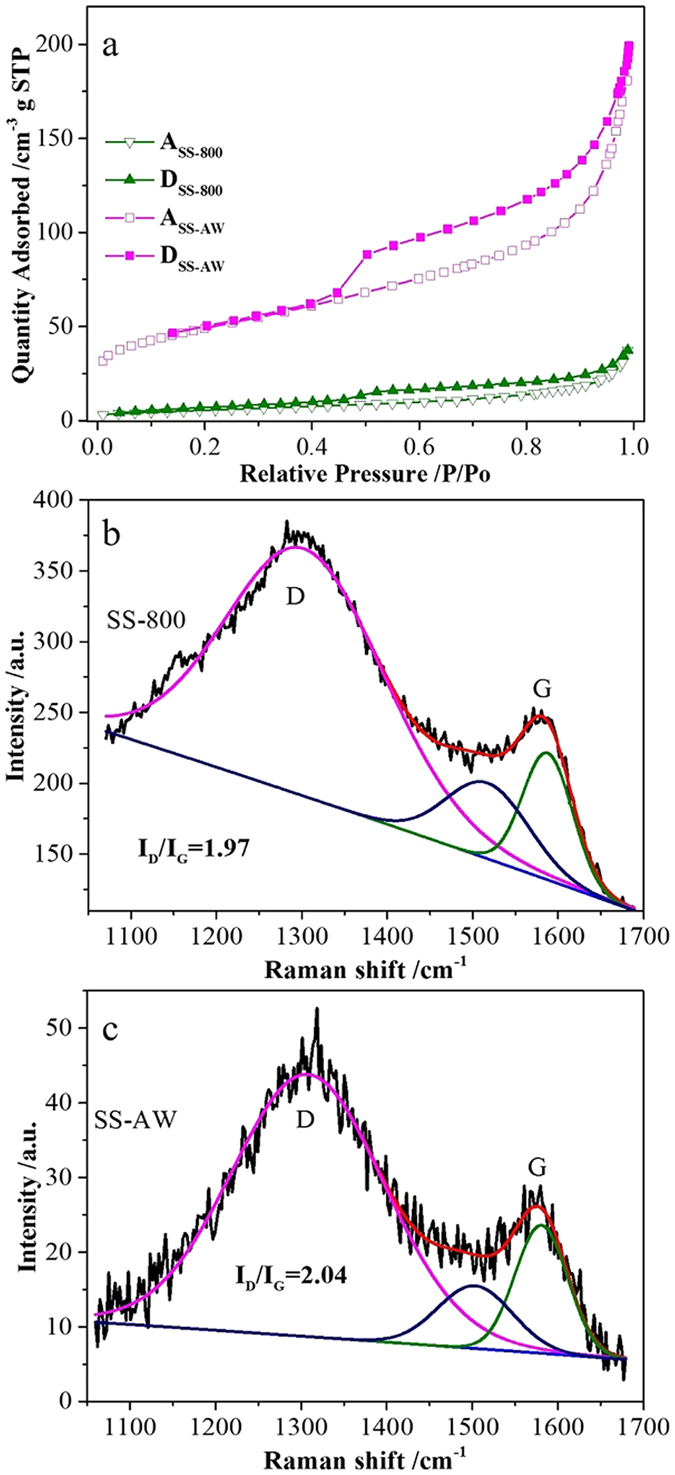
N_2_ adsorption–desorption isotherms (**a**) (A represents absorption and D represents desorption) and Raman spectra of the SS-800 and SS-AW (**b–d**).

The surface area and pore structure were assessed on the basis of the nitrogen adsorption-desorption isotherms. The isotherm curves of SS-800 and SS-AW show the type IV feature (according to the IUPAC classification), revealed the typical mesoporous features of the sewage sludge-derived nanomaterials (Fig. 2a)^24^,^25^. The upward trend of the isotherms at a low relative pressure (*P*/*P*_*o*_ < 0.4) implied the presence of micropores in the nanomaterials^24^,^25^, while the upward-type H3 hysteresis loop at *P*/*P*_*o*_ > 0.4 suggest that SS-800 and SS-AW were rich in mesopores. The slight upward tendency before 1.0 could be ascribed to the internal macropores between the accumulation particles in the nanomaterials, which could be observed in the SEM (Fig. 1b,d). The BET surface area and total pore volume of SS-AW is 390.14 m^2^ g^−1^ and 0.69 cm^3^ g^−1^, respectively, much higher than that of SS-800 due to the etch-out of the ash content. This surface area increase was accompanied by an increase in the average pore size from 3.83 to 6.89 nm, due to the mesopores generated during the acid washing process (Table 1 and Figure S4). The pore size distributions curves, calculated from the desorption branch by the Barrett-Joyner-Halenda method, further revealed the hierarchically mesoporous structure of SS-800 and SS-NC (Figure S4). The intrinsically rough morphology, favorable surface area, and hierarchically porous structure could largely expose catalytic active sites and effectively improve the absorption and reduction of oxygen molecules, thus facilitating the accessibility of the reactant for ORR^13^,^17^.

### Molecular structures and surface chemical compositions of the as-synthesized catalysts

Raman spectra were used to probe the molecular structures of the as-synthesized catalysts (Fig. 2b). Two characteristic peaks of carbon nanomaterial were clearly observed on both curves of the SS-800 and SS-AW, with the vibration band at about 1530 cm^−1^ assigned to the amorphous graphitic phase. The D peak, centered at 1294 cm^−1^, reflected the degree of disorder, and the G peak, centered at 1580 cm^−1^, reflected the level of graphitization^26^,^27^. *I*_*D*_*/I*_*G*_, the ratios of integrated intensity of the D and G band ratios, is widely used to assess the density of defects in graphite materials. The *I*_*D*_*/I*_*G*_ ratios of SS-800 and SS-AW, obtain from the peak height of the D and G band peak, were 1.97 and 2.04, respectively, indicating that the framework structures and functional groups were analogous to each other before and after the acid washing process (Fig. 2c,d). Thus, the acid washing process only etched out the ash content, rather than changing the carbon network of the as-synthesized catalysts. The much larger *I*_*D*_*/I*_*G*_ ratios for the as-synthesized catalysts may be caused by the insertion of heteroatoms into the carbon framework, thereby resulting in a defective structure^26^,^27^.

**Figure 3:**
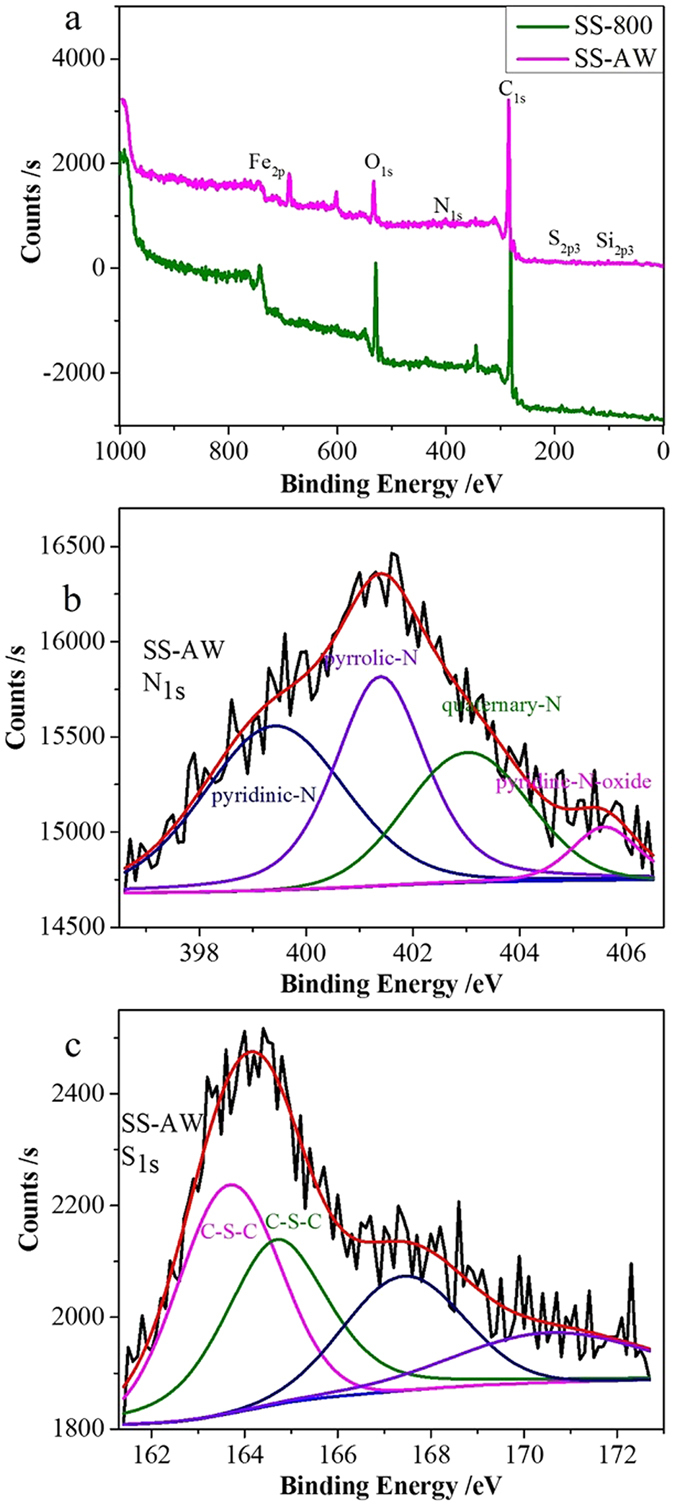
X-ray photoelectron spectra of the as-synthesized SS-800 and SS-AW (**a**). The high resolution N 1s and S 1s XPS spectra of the SS-AW are shown in (**b,c**), respectively.

XPS analysis was used to further examine the surface chemical compositions of the as-synthesized catalysts (Fig. 3). Obvious C 1s and O 1s peaks and fine N 1s, S 2p, Si 2p, and Fe 2p peaks were all observed, which is consistent with the results of elemental analysis, EDX, and ICP (Fig. 3a and Table 1). The C atoms were formed from the organic materials in the sewage sludge, which were subject to carbonization and graphitization during the pyrolysis process under the catalytic action of the critical toxic heavy metals uniformly distributed in the sewage sludge^28^. The N, S, and Fe atoms mainly originated from the intrinsic organic and inorganic compounds, such as proteins and iron salts, in the sewage sludge abundant in N, S, and Fe, while the Si atom derives from the residual SiO_2_. After the acid washing process, the contents of C increased, due to the etch-out of the ash content. The wishing process could remove most of the unwanted elements (Table 1). The correspondingly increase in N content clearly indicated the doped N atoms in the as-synthesized catalysts. The relative levels of S and Fe decrease with the leaching of the soluble S and Fe compounds, while the retained S and Fe elements appeared to covalently bond in the bulk of the material, instead of on the surface.

High-resolution scans of C, N, and S were performed and de-convoluted to obtain the corresponding atom binding states (Figs 3 and S5). In the case of the C1s XPS spectrum, the three peaks at 284.4, 286.0, and 288.4 eV were attributable to the sp^2^-hybridized graphitic carbon C-C, C-O/N/S, and C = O configurations, respectively (Figure S5a)^26^,^27^. The characteristic C-N/S bonds peaks found in the as-synthesized catalysts further confirmed that N and S heteroatoms were *in-situ* doped into the carbon framework during the pyrolysis process. The high-resolution N 1s peak of the as-synthesized catalyst fits into four types of nitrogen-containing groups at about 399.4, 401.4, 403.0 and 405.5 eV (Fig. 3b)^13^,^14^,^26^, which are typically observed in N-doped carbons. The peak located at about 399.4 eV was attributed to pyridinic N species, which was obtained by doping at the edge of the graphene layer. The peak located at 403.0 eV was attributed to quaternary N, which was the result of in-plane doping. The pyrrolic N species located at about 401.4 eV was assigned to nitrogen atoms in a pentagon structure, while the nitrogen species with a high binding energy (405.5 eV) can be assigned to oxidized nitrogen^13^,^14^,^26^. The doped N species, particularly the pyridinic and quaternary N species acting as n-type carbon dopants with the content of 36.6% and 23.5%, respectively, assist in the high surface polarity and fast charge-transfer rate which are favorable to proton and electron transfer for the ORR.

High-resolution S 2p XPS spectra was also used to analyze the sulfur doping in SS-AW (Fig. 3c). The two characteristic thiophene-S peaks located at 163.7 eV and 164.7 eV further indicate that S atoms were successfully doped into the carbon framework^13^,^14^. The presence of O originates from the possible residual oxygen-containing groups in the organic precursors (Figure S5b), while the identified Fe^3+^ in the bulk (Figure S5c) further implied the *in-situ* self-doped iron atoms into the carbon framework of the as-synthesized catalyst^13^. Thus it can be concluded that the pyrolysis process causes the *in-situ* N, Fe, and S self-doped into the carbon framework of the as-synthesized catalyst, and leads to the formation of pyridinic and quaternary N species and thiophene-S species which act as the electrocatalytic active sites^13^,^14^,^26^.

### Electrocatalytical activities

**Figure 4:**
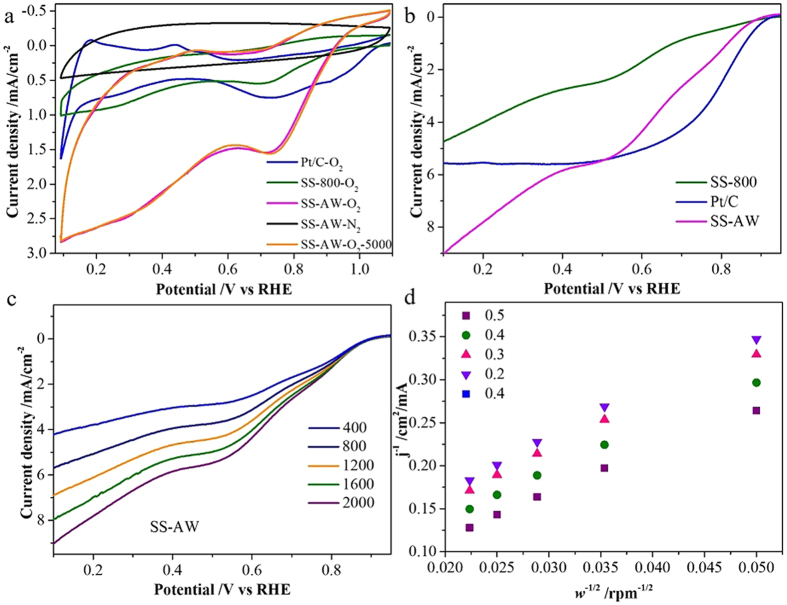
CVs curves of the as-synthesized SS-800, SS-AW and commercial Pt/C in N_2_ or O_2_ saturated 0.1 M KOH solutions with a scan rate of 50 mV s^−1^ (**a**). The SS-AW-O_2_-5000 represents the CV of SS-AW after continuous cycling of 5000 cycles. LSV curves of SS-800, SS-AW, and commercial Pt/C catalysts in O_2_-saturated 0.1 M KOH solutions at the scan rate of 5 mV s^−1^ with the rotation speed of 2000 rpm (**b**). LSV curves of SS-AW with different rotation speed in O_2_-saturated 0.1 M KOH solutions at the scan rate of 5 mV s^−1^ (**c**), and the K-L plots of SS-AW compared with commercial Pt/C (**d**).

CV measurements were carried out to investigate the electrocatalytic ORR activities of the as-synthesized catalysts (Fig. 4a). For the SS-AW, no obvious redox peak except a quasi-rectangular voltammogram, the typical supercapacitance effect of nanoporous carbon material, was obtained under N_2_ saturated solutions. In contrast, a well-defined characteristic ORR peak centered at about 0.72 V, which is close to commercial Pt/C (0.73 V) and SS-800, was observed under O_2_-saturated conditions, indicating a facile ORR process on this sewage sludge-derived *in-situ* self-doped nanoporous carbon material (Fig. 4a). Compared with that of SS-800, the larger peak current density of SS-AW is attributable to a synergetic combination of more crumpled morphology, larger surface area, and higher pore volume, which can largely expose catalytically active sites and enhance the O_2_ transport within the catalyst layer, thus facilitating the accessibility of the reactant for ORR^10^,^11^,^13^. In addition, the larger peak current density of SS-AW compared with the commercial Pt/C further suggests that the self-doped N, Fe, and S would be very effective in enhancing ORR activity and creating larger numbers of active sites. The almost identical CV curves of SS-AW electrode before and after the 5000 cycles confirmed the long-term operation stability of the as-synthesized sewage sludge-derived carbon-based catalyst.

LSV measurements were performed on an RDE to gain further insights into the ORR catalytic activity of the as-synthesized nanoporous carbon materials. The onset potential and current density for the SS-AW in O_2_-saturated 0.1 M KOH solutions were 0.89 V vs. RHE and 5.62 mA cm^−2^ at 0.45 V vs. RHE, both of which were comparable to those of 20 wt % Pt/C (0.91 V vs. RHE and 5.59 mA cm^−2^ at 0.4 V vs. RHE, respectively), suggesting an excellent electrocatalytic activity of the ORR in alkaline media (Fig. 4b). Typically, as the potential became more negative the current density simultaneously increased. It can be clearly observed that SS-800 has a higher limiting current density than that of nanoporous carbon materials derived at other pyrolysis temperatures (Figure S6a). With the acid washing process, the greatly increased limiting current density of SS-AW, which is comparable to that of commercial Pt/C, strongly indicates the excellent catalytic activity of ORR in the alkaline environment and further supports the CV observations described above.

LSV measurements performed on the RDE under different rotation speeds ranging from 400 rpm to 2000 rpm were recorded to gain further insight into the kinetics of the ORR at the SS-AW electrode, and for comparison with the commercial Pt/C electrode (Figs 4c and S6b). The limiting current density increases with the increasing rotation rate, verified the intrinsic diffusion-controlled ORR. The transferred electron number (n) per oxygen molecule can be calculated by the slopes of the K-L plots^13^,^26^. The plots of the inverse of limiting current density (*j*^−1^) versus the inverse of the square root of rotation speed (*ω*^−1/2^) for SS-AW and commercial Pt/C both exhibited favorable linearity at potentials ranging from 0.2 to 0.5 V versus the RHE electrode (Fig. 4d). The n for SS-AW calculated from the slope of the K-L plots was 3.26–3.66, which is close to the commercial Pt/C of 4.0, indicating that this sewage sludge-derived *in-situ* self-doped nanoporous carbon material dominantly proceeds with an almost four-electron reduction pathway for ORR, which is similar to the commercial Pt/C catalyst. Therefore, the superior ORR properties of SS-AW are highly desirable to reduce O_2_ almost entirely via a four-electron pathway, obtaining maximum energy capacity with a high current^13^,^14^,^26^.

**Figure 5:**
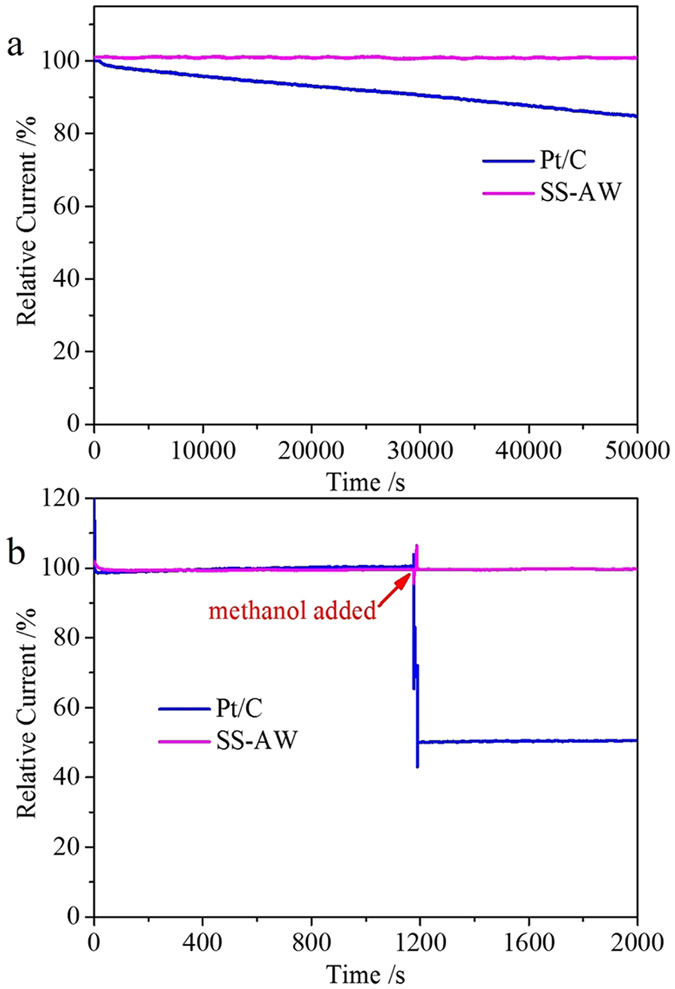
Chronoamperometric responses of SS-AW and commercial Pt/C catalyst at −0.30 V in O_2_-saturated 0.1 M KOH solutions at a rotation rate of 1600 rpm (**a**) and with 3 M methanol added after around 1000 s (**b**).

As the stability of the catalysts and the resistance to methanol crossover are two major concerns for practical application to fuel cells, the SS-AW was further assessed by the chronoamperometry approach, and compared with the commercial Pt/C catalyst (Fig. 5). A 13.9 h test of SS-AW under constant potentials in O_2_-saturated 0.1 M KOH aqueous solutions showed no obvious catalytic current density attenuation, compared with a 17% decrease in the commercial Pt/C under the same conditions, indicating the superior stability of SS-AW over the Pt/C catalyst (Fig. 5a). This higher stability can be attributed to the strongly bonded heteroatoms in the carbon framework, particularly after the acid washing process. The strength of the covalent heteroatom-carbon bonds in SS-AW could improve its chemical stability and effectively prevent the loss of active sites^13^,^14^. The methanol crossover is different from that of the Pt/C catalyst, which suffers from a sharp 49% decrease in current after the addition of 3 M methanol, while the catalytic current density of the SS-AW only show a subtle declination (Fig. 5b). These results indicate that the sewage sludge-derived *in-situ* self-doped nanoporous carbon material SS-AW catalyst exhibits excellent durability and outstanding immunity toward methanol crossover effects, thus having potential as an ideal ORR electrocatalyst that can be practically applied in direct methanol fuel cells.

**Figure 6:**
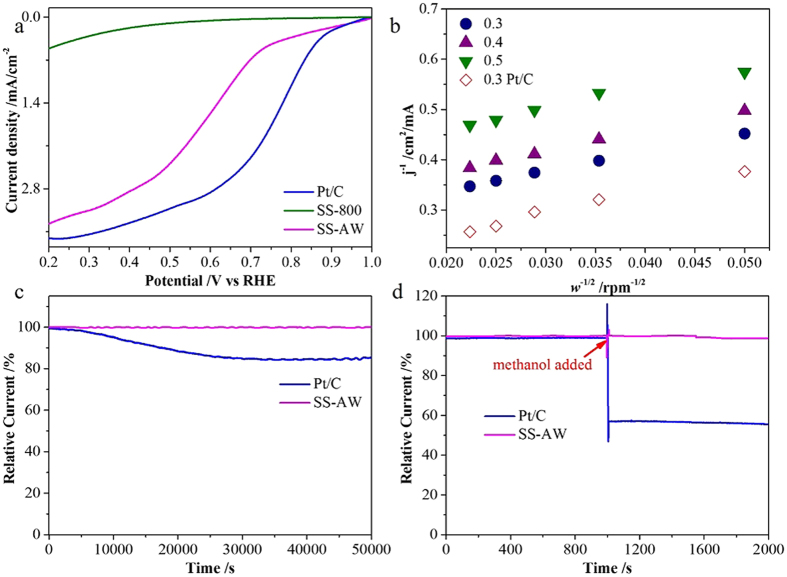
LSV curves of SS-800, SS-AW, and commercial Pt/C catalysts in O_2_-saturated 0.5 M H_2_SO_4_ solutions at the scan rate of 5 mV/s with the rotation speed of 2000 rpm (**a**), the K-L plots of SS-AW compared with commercial Pt/C (**b**), Chronoamperometric responses of SS-AW and commercial Pt/C catalyst at 0.30 V in O_2_-saturated 0.5 M H_2_SO_4_ solutions at a rotation rate of 2000 rpm (**c**) and with 3 M methanol added after around 1000 s (**d**).

Furthermore, the SS-AW also exhibited favorable electrocatalytic activity of the ORR in acidic media (Fig. 6), with an onset potential (0.94 V) and reduction current (3.35 mA cm^−2^ at 0.20 V vs. RHE) a little lower to those of the commercial Pt/C (0.95 V and 3.61 mA cm^−2^ at 0.20 V vs. RHE, respectively) in an O_2_-saturated 0.5 M H_2_SO_4_ solution (Fig. 6a). The calculated n for SS-AW in acidic environment were 3.17–3.52 (Fig. 6b), which is slightly lower than those of the Pt/C catalyst (4.0), reveals it almost four-electron reduction pathway for ORR^13^,^14^,^26^. Fig. 6c shows that the SS-AW has a better stability than the Pt/C in acidic environment, while the excellent tolerance to methanol poisoning effects in acidic environment was exhibited in Fig. 6d.

## Discussion

A unique heteroatom (N, Fe, and S) doped nanoporous carbon material, SS-AW, was synthesized via a facile direct pyrolysis process with sewage sludge acting as the “all-in-one” precursor. The as-synthesized nanoporous carbon material, which proceeds dominantly with an almost four-electron reduction pathway for ORR, exhibited favorable catalytic activity in both alkaline and acidic environments, with excellent durability and outstanding immunity to methanol crossover effects in alkaline environments. The excellent performance of this carbon nanomaterial could be attributed to the synergistic effect of the *in-situ* N, Fe, and S self-doped into the carbon framework, which act as active sites of ORR, and the hierarchically porous structure facilitating mass transport.

The inconspicuous limiting current density is due to the changed O_2_ penetration depth inside the electrode structure along with the changed potential^29^,^30^. For the Pt/C electrocatalyst, the O_2_ reduction is fast enough at high overpotential, thus the O_2_ reduction reaction is limited only on the outer part of the porous electrode, and a flat limiting plateau is observed. For the carbon based electrocatalysts, the distribution of electrocatalytic sites on the electrode surface is less uniform and the O_2_ reduction reaction is relatively slower, then some of the electrocatalyst particles inside the electrode might be in contact with O_2_ even at high overpotential. Thus no obvious current density plateau can be observed^29^,^30^.

The relatively lower catalytic activity of the as-synthesized SS-AW in H_2_SO_4_ solution may be due to quinine system of the carbon based electrocatalysts which is capable of reducing oxygen catalytically only in a strong base media^31^,^32^. It has been proposed that the semiquinone radical anion of the quinine systems is responsible for the high oxygen reduction activity of carbon materials in the solutions of high pH. In the solutions of low pH the semiquinone radical is protonated and in this case there is no evidence for the electrocatalysis of the oxygen reduction process^31^. Quantum chemical calculations further confirmed these experimental observations^32^.

The sewage sludge, which consists of organic material, mainly bacterial cells and bio-macromolecules, and inorganic components in the form of silica, iron salts, calcium oxide, alumina, magnesium oxide, and a wide variety of transition metals, act as a “all-in-one” precursor during the pyrolysis process^18^,^19^,^20^. The chemical contamination in the sewage sludge have important influence on this preparation. The special carbon framework of the as-synthesized catalyst stemmed from the carbonization and graphitization of organic materials in the sewage sludge under the catalytic action of the critical toxic heavy metals, with an in-built inorganic template to prevent agglomeration during the pyrolysis process. The special component of N, Fe, and S compounds innate in the sewage sludge, such as proteins and iron salts, worked as an ideal precursor and nature dopant for the *in-situ* N, Fe, and S self-doped into the carbon framework of the as-synthesized catalyst. All those type of chemicals, which were properly provided for the synthesis of this efficient and stable electrocatalyst, were the usually composed of sewage sludge. Thus, although the chemical contamination in the sewage sludge would vary from one wastewater treatment plants to another, the sewage sludge-derived catalyst in this research could be taken as a representative materials.

The acid washing step not only increased the electrocatalytic active contents, the surface area, and the total pore volume of the catalyst, but also enhanced the formation of the intrinsically rough morphology and the hierarchically porous structure of the catalyst, which could largely expose catalytic active sites and effectively improve the absorption and reduction of oxygen molecules, thus facilitating the accessibility of the reactant for ORR. Furthermore, the leaching of solubility compounds also ensured the high stability of the SS-AW.

Accordingly, the unique *in-situ* N, Fe, and S self-doped nanoporous carbon material we synthesized via direct pyrolysis of the “all-in-one” precursor sewage sludge exhibited favorable electrocatalytic activity, with excellent stability and durability. The efficient electrocatalytic properties of the SS-AW may be attributable to the combined effects of the intrinsically rough morphology, favorable surface area, and hierarchically porous structure, which largely exposes catalytically active sites and effectively improves the absorption and reduction of oxygen molecules. The synergistic effect of the *in-situ* N, Fe, and S self-doped into the carbon framework also acted as electrocatalytically active sites for ORR^13^,^14^,^26^. In other words, the unique qualities of this carbon nanomaterial and it excellent catalytic activity of ORR stem from the particular composition of sewage sludge and the proper utilization of almost all the components during the synthesized process.

Although there are several carbon nanomaterials generated for natural biomass been reported^13^,^14^,^26^, to the best of our knowledge, this is a rare report on cost-effective fabrication of multi-doped nanoporous carbon material without activation from waste/pollution as an efficient electrocatalyst for ORR in both alkaline and acidic environments. The levels of sewage sludge worldwide are increasing, with about 30 million tons in China, 74 million tons in Japan, 5.6–7 million dry tons in the US, and 9.8 million dry tons in the EU produced annually. The development of more cost-effective and environmentally benign value-added reuse of sewage sludge is therefore of particular concern^33^,^34^. Our protocol demonstrates the utilization of a proven pyrolysis technique, which is easy to handle and suitable for large-scale industrial production, to incrementally convert sewage sludge into an excellent electrocatalyst for ORR. Considering that most other doped carbon nanomaterials are derived from multiple hazardous chemicals precursors, our approach of developing high activity *in-situ* self-doped carbon-based nanomaterials from waste/pollution via facile methods suitable for large-scale production deserves particular attention.

## Methods

### Preparation of the sewage sludge-derived nanoporous carbon material

A dewatered sewage sludge sample, as the precursor for the process, was obtained from the Anting wastewater treatment, located in Shanghai, China^24^. All reagents were of analytical grade and were used as received.

The sewage sludge-derived heteroatom-doped nanoporous carbon material was synthesized by a facile one-step pyrolysis process in a quartz tube furnace (Figure S1). In brief, the sewage sludge was first freeze-dried to remove the water, and the lyophilized sewage sludge was placed in a quartz tube furnace under 100 ml min^−1^ of N_2_ flow for 30 min to completely remove the air. The furnace was heated to the 800 °C at a heating rate of 5 °C min^−1^ under a 50 ml min^−1^ of N_2_ flow, and maintained at that temperature for 2 h. After being naturally cooled to ambient temperature in the N_2_ flow, the resultant black powder was obtained and denoted as SS-800. The SS-800 was immersed in 20 wt% HF to etch-out the SiO_2_, leached in 20 wt% HCl at 80 ◦C for 2 hour three times to remove any soluble impurity, then washed until the pH reached 7, and dried at 100 °C to obtain the sewage sludge-derived nanoporous carbon material (SS-AW).

### Characterization

Scanning electron microscopy (SEM; EDAX XL30, The Netherlands) was carried out to image the morphology of the as-synthesized sewage sludge-derived nanomaterials. Nitrogen sorption/desorption measurements were taken with an AUTOSORB-IQ instrument (Quantachrome Co., USA) to analyze the Brunauer-Emmett-Teller (BET) surface area and the pore size distribution of the nanomaterials. X-ray diffraction (XRD) patterns were measured (X’Pert PRO, Philips Co., The Netherlands) to determine the crystal structure of the nanomaterials. The Raman spectra were determined by a laser Raman spectrometer (SPEX/403, JY., France), with a 532 nm laser excitation. The surface electronic environment of the nanomaterials was investigated by X-ray photoelectron spectroscopy (XPS, PHI-5000C, Perkin-Elmer Co., USA). The composition of the as-synthesized carbon nanomaterials was measured via an energy-dispersive X-ray spectroscopy (EDX) system directly and an inductively coupled plasma spectrometry (ICP, Agilent 720ES, USA) after total digestion in a microwave using a mixture of HNO_3_ + HCl + HF. The contents of C, N, and O were also determined by an Elemental Analyzer system (vario EL III, GmbH, Germany).

### Electrochemical characterization

All the electrochemical tests were carried out on a CHI760E electrochemical workstation (Shanghai Chenhua Instruments Co., China) connected to a rotating disk electrode (RDE) system (Pine Research Instrumentation Inc., USA) at room temperature. 0.1 M KOH solution and 0.5 M H_2_SO_4_ solution were used as the alkaline and acid electrolyte, respectively, and were saturated with N_2_ or O_2_ for 30 min prior to electrochemical testing. A graphite rode was used as the counter electrode and an Hg/HgO and Hg/HgSO_4_ electrode were used as the reference electrode in the alkaline and acid electrolyte, respectively. The working electrodes were prepared by dropping the catalyst ink onto a glassy carbon disk (5.0 mm in diameter) substrate. For each sample, 6.0 mg of the as-synthesized sewage sludge-derived nanoporous carbon material or commercial 20 wt % Pt/C catalyst (Alfa Aesar) was ultrasonically dispersed in 3 ml of ethanol for 30 min. Then, 10 μL of this uniform, well-dispersed ink was dropped on the glassy carbon electrode and dried at room temperature. A 5 μL solution of Nafion (0.05 wt %) was then pipetted on the surface of the catalyst layer and dried at room temperature to form a protective film.

Cyclic voltammetry (CV) curves were obtained in N_2_ or O_2_ saturated electrolyte solutions with a scan rate of 50 mV s^−1^. Before the measurements, all the CV curves were repeatedly swept in O_2_ saturated 0.1 M KOH solutions until a steady CV curve was obtained. Linear sweep voltammetry (LSV) measurements were conducted in O_2_ saturated electrolyte solutions with rotating speeds varying between 400 rpm to 2000 rpm at a rate of 5 mV s^−1^. Methanol crossover and durability tests were conducted using the chronoamperometric technique in O_2_ saturated electrolyte solutions with a rotation rate of 2000 rpm. The Koutecky-Levich (K-L) equation was utilized to calculate the transferred electron number (n) at various electrode potentials^13^,^17^. For comparison, the ORR activity and stability of a commercial Pt/C catalyst were also measured following the same procedures under the same conditions.

## Additional Information

**How to cite this article**: Yuan, S.-J. and Dai, X.-H. Facile synthesis of sewage sludge-derived *in-situ* multi-doped nanoporous carbon material for electrocatalytic oxygen reduction. *Sci. Rep*. **6**, 27570; doi: 10.1038/srep27570 (2016).

## Supplementary Material

Supplementary Information

## Figures and Tables

**Table 1:** Properties of the as-synthesized sewage sludge-derived catalysts.

	SS-800	SS-AW
*S*_BET_ (m^2^/g)	29.92	390.14
average pore size (nm)^a^	3.83	6.89
conc. (wt %)		
C^b^	36.57	56.16
N^b^	0.77	1.38
S^c^	0.88	0.45
Fe^d^	14.10	6.14
Si^c^	2.94	0.20
Al^d^	1.56	0.01
Mg^d^	4.29	0.02
Ca^d^	0.89	0.02
Cr^d^	0.05	–
Mn^d^	0.40	–
Ni^d^	0.10	–
Cu^d^	0.13	–

^a^Calculated from the Barrett-Joyner-Halenda equation using the desorption isotherm.

^b^The contents of C, N, and S were obtained by Elemental Analyze.

^c^The content of Si was obtained by EDX.

^d^The contents of metals were obtained by ICP.
