# Supplementary material for: Facile synthesis of sewage sludge-derived in-situ multi-doped nanoporous carbon material for electrocatalytic oxygen reduction
Source: Sci Rep. 2016 Jun 7;6:27570. doi: 10.1038/srep27570 (PMC4895130; doi:10.1038/srep27570)
Supplement: Supplementary Information [file srep27570-s1.doc]

**Electronic Supplementary Information for**

**Facile synthesis of** **sewage sludge-derived** ***in-situ* multi-doped** **nanoporous carbon material as an efficient** **electrocatalyst for oxygen reduction**

Shi-Jie Yuan, Xiao-Hu Dai*

State Key Laboratory of Pollution Control and Resource Reuse, College of Environmental Science and Engineering, Tongji University, Shanghai, 200092, China

***Corresponding author:**

Prof. Xiao-Hu Dai, Phone: +86-21-65986297; Fax: +86-21-65983602; E-mail: [daixiaohu@tongji.edu.cn](mailto:daixiaohu@tongji.edu.cn)


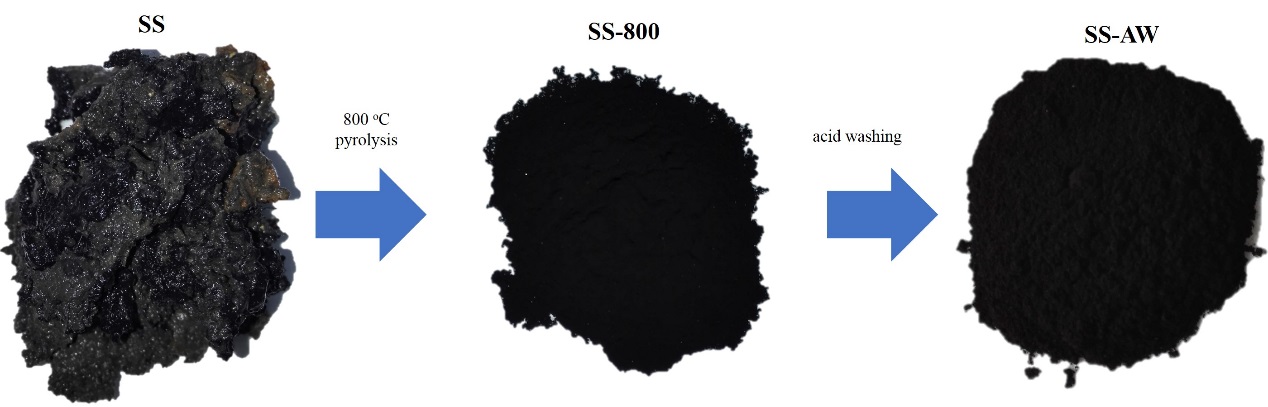


Fig. S1 The schematic presentation of the synthesis process.


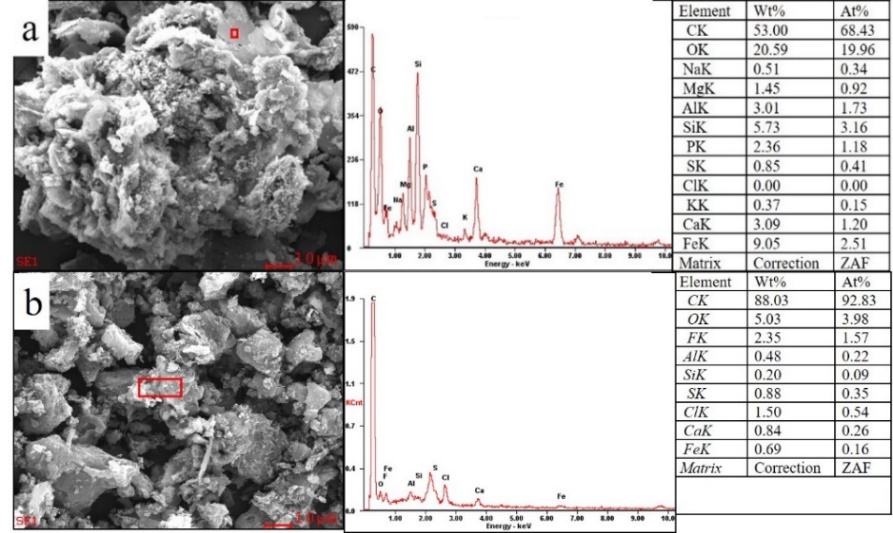


Fig. S2 Energy dispersive X-ray (EDX) spectra of the SS-800 (a) and SS-AW (b).


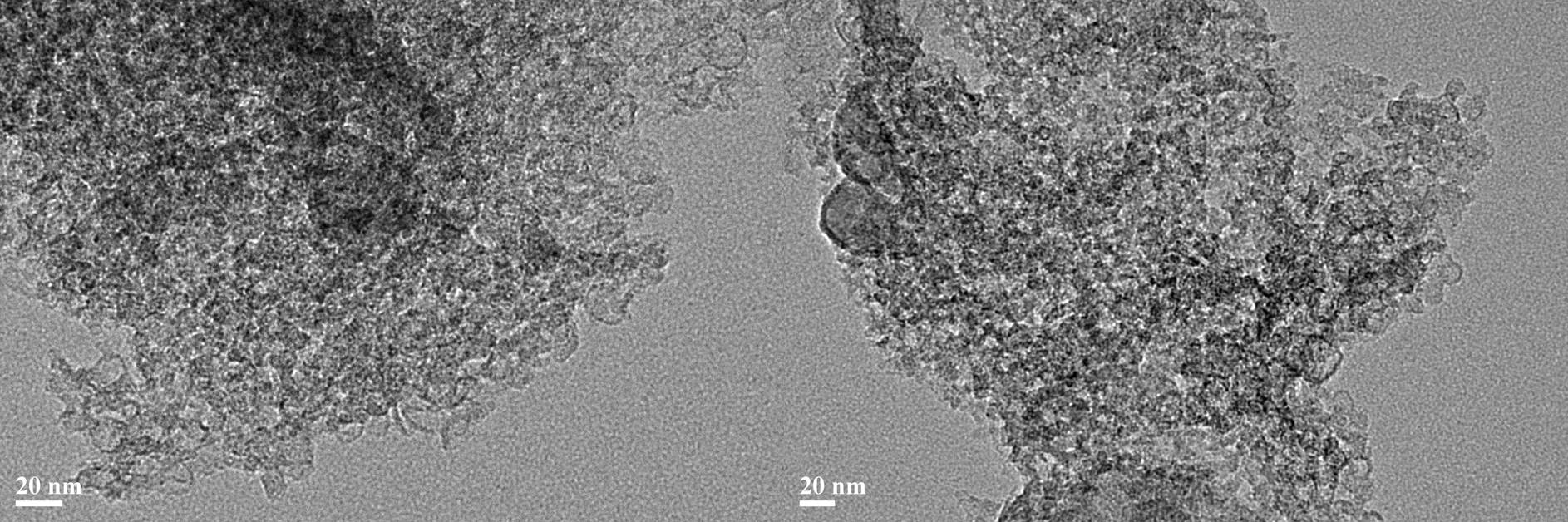


Fig. S3 Typical TEM images of the SS-AW.


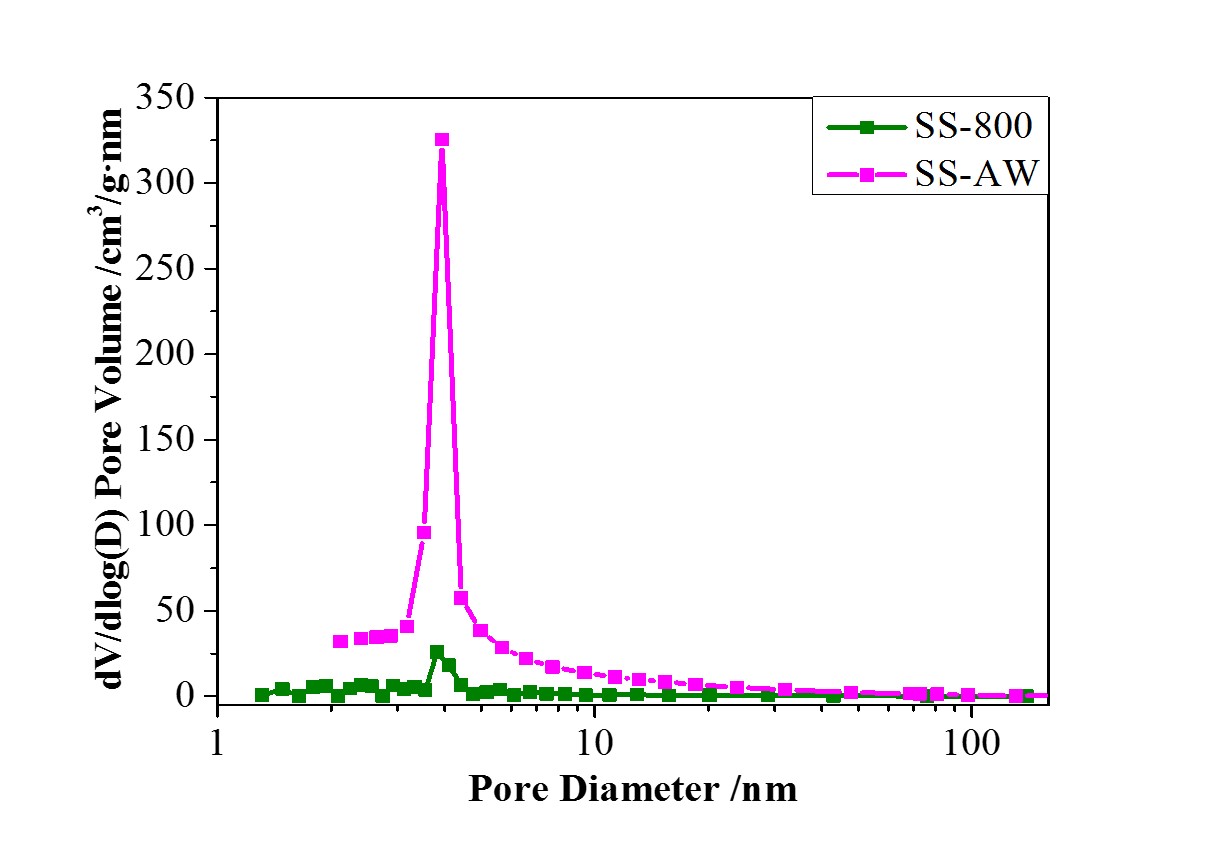


Fig. S4 Pore size distributions of the SS-800 and SS-AW.


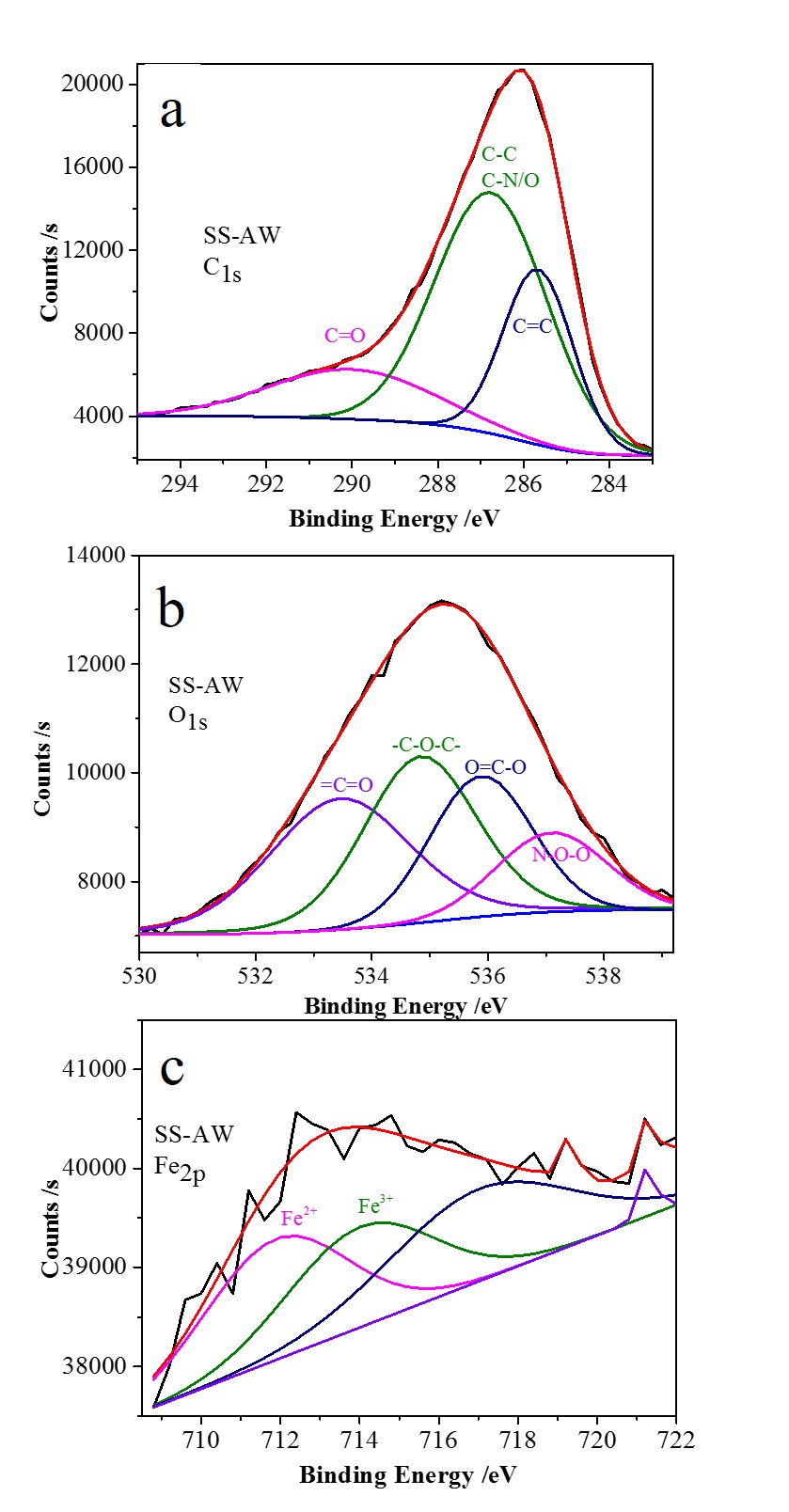


Fig. S5 The high resolution C 1s (a), O 1s (b), and Fe 1s (c) XPS spectra of the SS-AW.


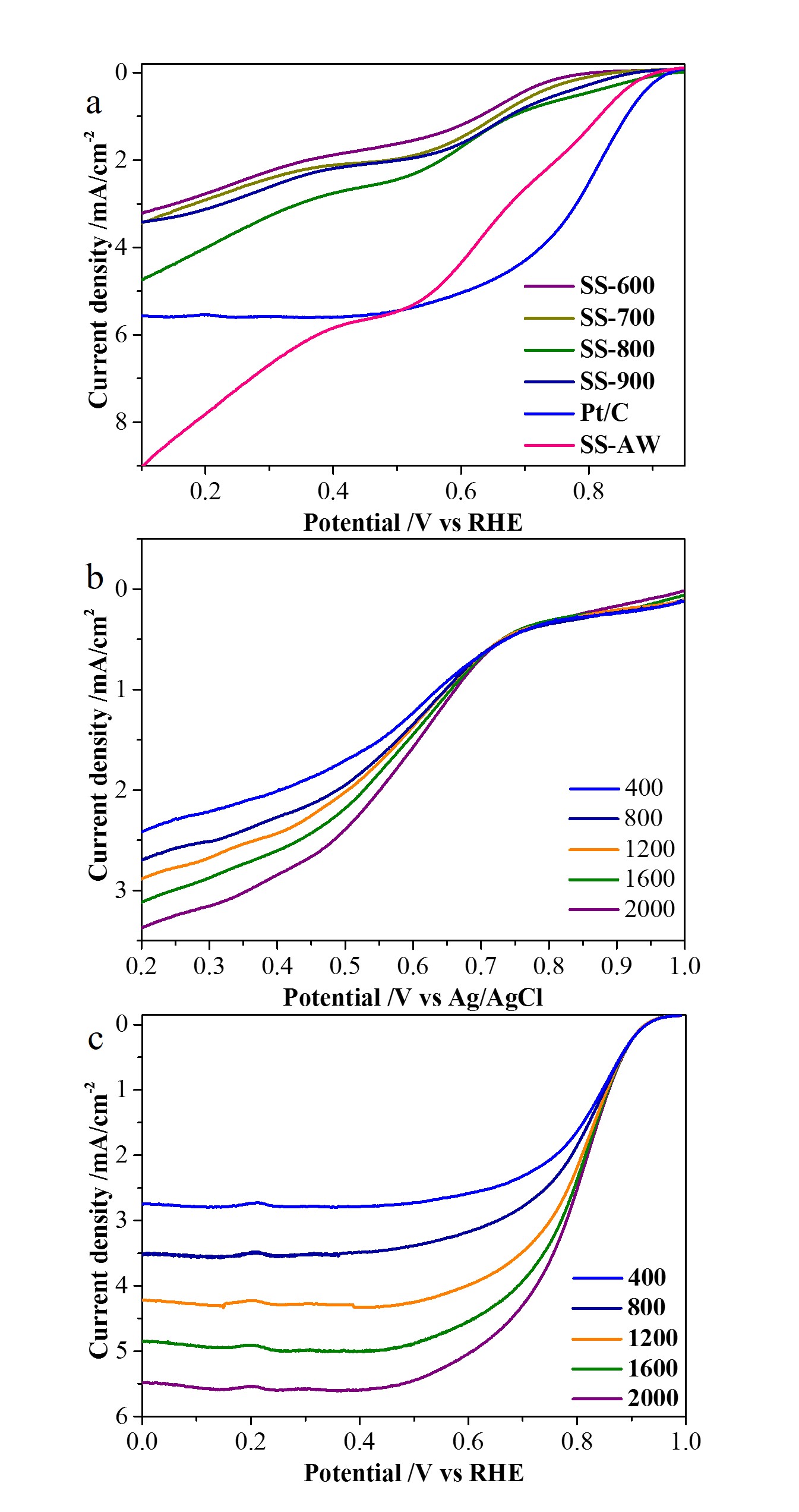


Fig. S6 LSV curves of SS-600, SS-700, SS-800, SS-900, SS-AW, and commercial Pt/C catalysts in O_2_-saturated 0.1 M KOH solutions at the scan rate of 5 mV s^-1^ with the rotation speed of 2000 rpm (a), LSV curves of SS-AW with different rotation speed in O_2_-saturated 0.5 M H_2_SO_4_ solutions at the scan rate of 5 mV/s (b), and LSV curves of commercial Pt/C catalysts with different rotation speed in O_2_-saturated 0.1 M KOH solutions at the scan rate of 5 mV/s (c).
